# Maximizing crossbred performance through purebred genomic selection

**DOI:** 10.1186/s12711-015-0099-3

**Published:** 2015-03-14

**Authors:** Hadi Esfandyari, Anders C Sørensen, Piter Bijma

**Affiliations:** Center for Quantitative Genetics and Genomics, Department of Molecular Biology and Genetics, Aarhus University, Aarhus, Denmark; Animal Breeding and Genomics Centre, Wageningen University, Wageningen, the Netherlands

## Abstract

**Background:**

In livestock production, many animals are crossbred, with two distinct advantages: heterosis and breed complementarity. Genomic selection (GS) can be used to select purebred parental lines for crossbred performance (CP). Dominance being the likely genetic basis of heterosis, explicitly including dominance in the GS model may be an advantage to select purebreds for CP. Estimated breeding values for CP can be calculated from additive and dominance effects of alleles that are estimated using pure line data. The objective of this simulation study was to investigate the benefits of applying GS to select purebred animals for CP, based on purebred phenotypic and genotypic information. A second objective was to compare the use of two separate pure line reference populations to that of a single reference population that combines both pure lines. These objectives were investigated under two conditions, i.e. either a low or a high correlation of linkage disequilibrium (LD) phase between the pure lines.

**Results:**

The results demonstrate that the gain in CP was higher when parental lines were selected for CP, rather than purebred performance, both with a low and a high correlation of LD phase. For a low correlation of LD phase between the pure lines, the use of two separate reference populations yielded a higher gain in CP than use of a single reference population that combines both pure lines. However, for a high correlation of LD phase, marker effects that were estimated using a single combined reference population increased the gain in CP.

**Conclusions:**

Under the hypothesis that performance of crossbred animals differs from that of purebred animals due to dominance, a dominance model can be used for GS of purebred individuals for CP, without using crossbred data. Furthermore, if the correlation of LD phase between pure lines is high, accuracy of selection can be increased by combining the two pure lines into a single reference population to estimate marker effects.

**Electronic supplementary material:**

The online version of this article (doi:10.1186/s12711-015-0099-3) contains supplementary material, which is available to authorized users.

## Background

One of the main limitations of many livestock breeding programs is that selection is carried out in purebred nucleus lines or breeds that are housed in high-health environments, whereas the goal of selection is to improve crossbred performance (CP) under field conditions. Due to genetic differences between purebred and crossbred animals and to environmental differences between nucleus and field conditions, performance of purebred parents can be a poor predictor of the performance of their crossbred descendants [1]. Several methods have been proposed as alternatives to pure line selection to obtain greater response in crossbred populations. These methods can be classified into three groups: reciprocal recurrent selection, combined crossbred and purebred selection (CCPS) and genomic selection (GS).

Numerous studies have provided encouraging results regarding the application of GS in purebred populations [2,3]. However, in livestock production systems, many animals are crossbred, with two distinct advantages i.e. heterosis and breed complementarity. Different GS models have been proposed and used to select purebred animals for CP [1,4]. Dekkers [1] demonstrated that marker-assisted selection or GS with marker effects derived at the commercial crossbred level can lead to substantially higher gain in CP and a lower rate of inbreeding compared to CCPS when marker effects were estimated accurately.

If one accepts that GS is an appropriate tool to select animals for CP, then another issue to solve is: should marker effects be estimated from purebred or crossbred animals? Using simulated data on training populations that consisted of crossed or mixed breeds, Toosi et al. [5] reported that the accuracy of GS by using crossbred data for training was lower than using purebred data for training, but not substantially lower. However, the GS model used in [5] assumed that single nucleotide polymorphism (SNP) allele effects were the same in all breeds. In crossbred populations, effects of SNPs may be breed-specific because the extent of linkage disequilibrium (LD) between SNPs and quantitative trait loci (QTL) can differ between breeds. SNP effects may also differ due to dominance and epistasis. Moreover, the LD may not be restricted to markers that are tightly linked to the QTL. Both these problems have been addressed by using a model with breed-specific effects of SNP alleles (BSAM) [1] and the performance of BSAM has been studied by stochastic simulations [4,6]. Under additive gene action, fitting BSAM was beneficial only when the parental breeds were distantly related and the number of SNPs was small relative to the size of the training population [4].

In most studies, additive gene action or perfect knowledge of allele substitution effects or both are assumed [4,5]. It has been argued that dominance is the likely genetic basis of heterosis [7], therefore explicitly including dominance in the GS model may be an advantage to select purebred animals for CP. With dominance, allele substitution effects and individual breeding values depend on allele frequency and, thus, change over time, which alters the ranking of individuals. This problem can be overcome by applying a dominance model, which provides estimates of both additive and dominance effects and, therefore, enables the computation of allele substitution effects using appropriate allele frequencies. Once SNP effects are estimated for the training population, they can be successively applied over generations with updated allele frequencies to develop prediction equations specific to a given generation [8]. Zeng et al. [8] compared additive and dominance models for GS of purebred animals for CP and came to the conclusion that, when dominance is the sole driver of heterosis, using a dominance model for GS is expected to result in greater cumulative response to selection of purebred animals for CP than either BSAM or the additive model. The extent of this additional response to selection depended on the size of dominance effects at the QTL and the power of detection of dominance effects through SNP genotypes. The results of [8] suggested that in the presence of dominant gene action, compared with BSAM and additive models, GS with a dominance model is better at maximizing CP through purebred selection, especially when no retraining is carried out at each generation.

Previous studies on the selection of purebred animals for CP [4,8] focused on crossbred data to estimate marker effects, which requires collecting genotypes and phenotypes on crossbred animals. This can substantially increase the required financial investment of the breeding program, since crossbred animals are usually not individually identified and individual performance is not recorded. It is interesting to evaluate the potential benefit of GS within purebred lines when the objective is to improve performance of crossbred animals, by using marker effects that are estimated from pure line data. In other words, additive and dominance effects of alleles can be estimated from pure line data, and subsequently breeding values for CP can be estimated by using the appropriate allele frequencies. Thus, our objective was to investigate the benefits of GS of purebred animals for CP based on purebred information and using dominance model, compared to traditional selection for purebred performance. A second objective was to compare the use of two separate pure line reference populations with that of a single reference population that combined the pure lines. These objectives were investigated under two conditions, i.e. either a low or a high correlation of LD between the pure lines.

## Methods

### Population structure

**Figure 1:**
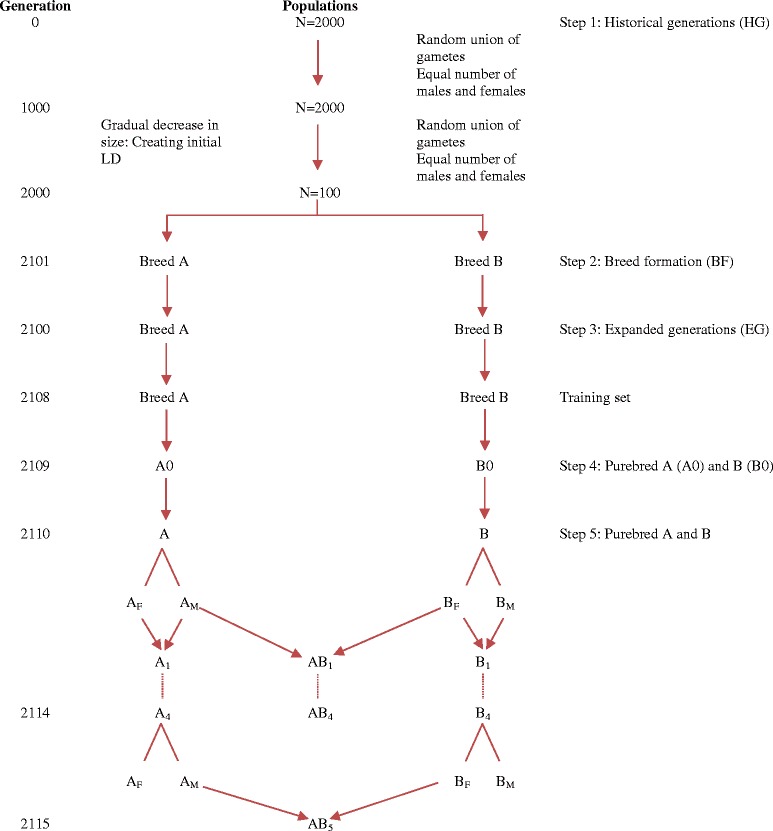
**Schematic representation of the simulation steps.** The crossbreeding program started in step 5 and consisted of five generations of purebred selection for crossbred performance; individuals from the last generation of step 3 (Generation 2108) constitutes the training population; A_*M*_ and B_*M*_ represent the males selected from breeds A and B, respectively; A_*F*_ and B_*F*_ represent the females selected breeds A and B, respectively; lines with arrows denote reproduction, while lines without arrows denote selection.

Using the QMSim software [9], a historical population was simulated forward in time. Subsequent generations, GS, and evaluation were simulated using a script developed in R version 2.15.2 [10] (Table 1 and Figure 1). In the first simulation step, 1000 discrete generations with a constant population size of 2000 were simulated, followed by 1000 generations with a gradual decrease in population size from 2000 to 100 in order to create initial LD. The number of individuals of each sex remained the same in this step and the mating system was based on random union of gametes that were randomly sampled from the male and female gamete pools. Therefore, only two evolutionary forces were considered in this step: mutation and drift. To simulate the two recent purebred populations (referred to as breeds A and B, hereafter), two random samples of 50 animals were drawn from the last generation of the historical population and each animal was randomly mated for another 100 generations (step 2).

**Table 1:** **Parameters of the simulation process**

**Population structure**	
**Step 1: Historical generations (HG)**	
Number of generations(size) - phase 1	1000 (2000)
Number of generations(size) - phase 2	1000 (gradual decrease)
Selection and mating	Random
**Step 2: Breed formation (BF)**	
Number of founder males from HG	50
Number of founder females from HG	50
Number of generations	100
Number of offspring per dam	5
Selection and mating	Random
Step 3*: Expanded generations (EG)	
Number of founder males from BF	100
Number of founder females from BF	100
Number of generations	8
Number of offspring per dam	10
Selection and mating	Random
**Step 4: Purebred A0 and B0**	
Number of founder males/females from EG breed A	100/200
Number of founder males/females from EG breed B	100/200
Number of offspring per dam	5
Mating system	Random
Selection and mating	Random
**Step 5: Purebred A and B**	
Number of males/females from A0	100/200
Number of males/females from B0	100/200
Number of offspring per dam	5
Selection	GEBV
Mating system	Random
Heritability of the trait	0.3
Phenotypic variance	1
**Genome**	
Number of chromosomes	1
Number of SNPs	1000
SNP distribution	Random
Number of QTL	100
QTL distribution	Random
MAF of SNPs	0.05
MAF of QTL	0.05
Additive allelic effects for SNPs	Neutral
Additive allelic effects for QTL	Gamma
Rate of recurrent mutation	2.5 × 10^−4^

*All of the individuals from the last generation of step 3 (Generation 8) was the training set.

In the next simulation step (step 3), in order to enlarge population size for breeds A and B, eight generations were simulated with ten offspring per dam. The mating within each breed was again based on random union of gametes and no selection was considered in this step. Within each breed, all animals in generation 8 of this step were considered as training population for the estimation of marker effects.

In the next step (step 4), for each breed, 100 males and 200 females were sampled randomly from the last generation of step 3 and mated randomly to produce 1000 purebred animals (A0 and B0). In the subsequent generations (step 5), a two-way crossbreeding program with five generations of selection was simulated, as illustrated in Figure 1. The goal was to improve CP through selection in the two parental breeds (breeds A and B acted as sire and dam breeds, respectively). The selection criterion in the purebred population was either the rank of the individual’s genomic estimated breeding value (GEBV) for purebred performance (GEBVP), or its GEBV for crossbred performance (GEBVC). SNP effects for the prediction of GEBV for each breed were estimated only once, using the purebred reference population of generation 8 of step 3 (these are the parents of generations A0 and B0). These estimates of SNP effects were then repeatedly applied to predict either GEBVP or GEBVC in the following five generations of selection of the pure breeds. In generation 1 through 5, 300 animals (the top 100 males and top 200 females) were selected from the 1000 available candidates in each parental breed, based on their GEBV. Thus, the selected proportions were 20% (100 out of 500) in males and 40% in females (200 out of 500). The selected animals were randomly mated within each breed to produce 1000 purebred replacement animals for the next generation. Meanwhile, the 100 selected males of breed A were randomly mated to the 200 selected females of breed B to produce 1000 crossbred progeny (step 5). The phenotypic mean of crossbred animals was computed for each generation of selection (AB_1_ to AB_5_) to evaluate the cumulative response to selection.

### Genome and trait phenotypes

A genome consisting of one chromosome of 1 Morgan with 100 segregating QTL and 1000 markers was simulated (Table 1). Both QTL and markers were randomly distributed over the chromosome. To reach the required number of segregating loci after 2000 generations, about two to three times as many bi-allelic loci were simulated with starting allele frequencies sampled from a uniform distribution and a recurrent mutation rate of 2.5 × 10^−4^. To build the SNP panel, 1000 SNPs were randomly drawn from segregating SNPs that had a minor allele frequency (MAF) of at least 0.05, in the last historical generation. The additive effect (*a*) of a QTL was defined as half the difference in genotypic value between alternate homozygotes and the dominance effect (*d*) as the deviation of the value of the heterozygote from the mean of the two homozygotes [7]. A gamma distribution with shape and scale parameters of 0.4 and 1.66, respectively, was used to generate the unsigned value of the additive effect for each QTL. This provided an L-shaped distribution of QTL effects. With equal probability, one of the two alleles was chosen to be positive or negative. Previous studies have not shown a consistent relationship between additive and dominance effects of QTL [11]. Similar to Wellmann and Bennewitz [12,13], we simulated relative dominance degrees *h*_*i*_ that were normally distributed, *N*(0.5, 0.1), and independent of the additive effects. Next, absolute dominance effects were *d*_*i*_ = *h*_*i*_.|*a*_*i*_| where |*a*_*i*_| is the absolute value of the additive effect. Thus, additive and dominance effects were dependent. Additive and dominance effects were scaled in each replicate of each scenario such that additive and dominance variances were equal to 0.3 and 0.1, respectively. This was done to ensure that each scenario had the same genetic variance, such that this could not contribute to differences among scenarios. After scaling, 10 to 15% of QTL showed overdominance. Trait phenotypes were simulated by adding a standard normal residual effect to the genotypic value of each animal. The variance of the residual effects was chosen such that broad-sense heritability *H*^2^ of the trait was equal to 0.4. As a result, phenotypic variance (${\sigma}_{\mathrm{p}}^2$) was 1, narrow-sense heritability *h*^2^ was equal to 0.3 and dominance variance was 0.1 ${\sigma}_{\mathrm{p}}^2$.

### Estimation of marker effects

The Bayesian LASSO proposed by Park and Casella [14] and developed by de los Campos et al. [15] was used to estimate marker effects. The difference between Bayesian LASSO and the Bayesian approaches developed by Meuwissen et al. [2] (BayesA and BayesB) stems from the specification of the *a priori* variance of the marker-specific regression coefficient. We used the BLR “Bayesian linear regression” R package developed by Perez et al. [16]. The following model was used to estimate the genetic effect associated with each marker:

$$ {y}_i=\mu +{\displaystyle \sum }{X}_{ij}{a}_j+{\displaystyle \sum }{Z}_{ij}{d}_j+{e}_i, $$

where *y*_*i*_ is the phenotypic value of individual *i* in the training data, *μ* is the overall mean, *X*_*ij*_ is the copy number of a given allele of marker *j*, coded 0, 1 and 2 for aa, aA and AA, respectively, *a*_*j*_ is the random unknown additive effect for marker *j*, *Z*_*ij*_ is the indicator variable for heterozygosity of individual *i* at marker *j*, with *Z*_*ij*_ = 0 when individual *i* is homozygous at marker *j* (aa or AA) and *Z*_*ij*_ = 1 if individual *i* is heterozygous at marker *j* (aA), *d*_*j*_ is the random unknown dominance effect for SNP *j*, and *e*_*i*_ is the residual effect for animal *i* and Σ denotes summation over all marker loci *j*.

The prior distribution of the residual variance was a scaled inverse *χ*^2^ such that ${\sigma}_e^2\sim {\chi}^{-2}\ \left(d{f}_e,\ {S}_e\right)$. The degrees of freedom (*df*_*e*_) and the scale parameter (*S*_*e*_) for residual variance were set at 3.5 and 3, respectively. The conditional prior distribution of the marker effects was a Gaussian distribution with prior variance specific to each marker: ${a}_j\sim N\ \left(0,{\sigma}^2\epsilon {\tau}_j^2\right)$ for *j* = 1,…, *m*, with *τ*_*j*_^2^ following an exponential prior distribution defined by ${\tau}_j^2\sim exp\ \left({\lambda}^2\right)$. The regularisation parameter *λ*^2^ followed a Gamma distribution, as suggested in [14]. In addition, an inverted Chi-square distribution was used for the variance of dominance effects: ${\sigma}_d^2\sim {\chi}^{-2}\ \left(d{f}_d,\ {S}_d\right)$ with *df*_*d*_ = 3 and *S*_*d*_ = 0.0005. The parameters of the prior distributions were computed according to the guidelines of the BLR package [15,16]. The BLR method used an MCMC algorithm to generate 10 000 samples, with the first 1500 samples discarded as burn-in.

### True and genomic estimated breeding values

Two types of true breeding values (TBV) were calculated, i.e. TBV for purebred performance (TBVP) and TBV for crossbred performance (TBVC). The TBV were calculated as the expected genotypic value of the offspring of a parent carrying a certain QTL-genotype, when this parent is mated at random to its own line (TBVP) or to the other pure line (TBVC). Thus, for animal *i* from breed *r*, the TBV for purebred performance was calculated as:

1 $$ TBV{P}_{ir}={\displaystyle \sum_{j=1}^{100}}\left[\left({x}_{ij}\right)\left({p}_{jr}{a}_j+{q}_{jr}{d}_j\right)\right]+\left[\left({y}_{ij}\right)\left(0.5{p}_{jr}{a}_j+0.5{q}_{jr}{d}_j+0.5{p}_{jr}{d}_j-0.5{q}_{jr}{a}_j\right)\right]+\left[\left({z}_{ij}\right)\left(-{q}_{jr}{a}_j+{p}_{jr}{d}_j\right)\right], $$

where *x*_*ij*_, *y*_*ij*_ and *z*_*ij*_ are indicator functions of the genotype of the *j*^*th*^ QTL of the *i*^*th*^ individual, with *x*_*ij*_ = 1 when the genotype is AA and otherwise 0, *y*_*ij*_ = 1 when the genotype is Aa or aA and otherwise 0, and *z*_*ij*_ = 1 when the genotype is aa and otherwise 0. Moreover, *p*_*jr*_ and *q*_*jr*_ are the allelic frequencies (A and a) for the *j*^*th*^ QTL in breed *r*, and *a*_*j*_ and *d*_*j*_ are true additive and dominance effects of the *j*^*th*^ QTL. For example, for an AA parent at locus *j*, a fraction *p*_*jr*_ of its offspring will have genotype AA, while a fraction *q*_*jr*_ of its offspring will have genotype Aa. Hence, for locus *j*, the breeding value of this parent equals (*p*_*jr*_*a*_*j*_ + *q*_*jr*_*d*_*j*_), which is the first term in Equation 1.

For crossbred offspring, the expected genotype frequencies of the offspring of a parent depend on the allele frequency in the other pure line (denoted *r’* here). Thus, for animal *i* from breed *r*, the TBV for CP was calculated using Equation 1, however *p*_*jr*_ and *q*_*jr*_ were replaced by *p*_*jŕ*_ and *q*_*jŕ*_, where *p*_*jŕ*_ and *q*_*jŕ*_ are the allele frequencies (A and a) for the *j*^*th*^ QTL in breed *r’*. We also calculated the correlation (*r*_*tbvp*,*tbvc*_) between TBVP and TBVC, which is known as the purebred-crossbred genetic correlation, denoted as *r*_*pc*_ by Wei and Vanderwerf [17].

Genomic estimated breeding values were calculated in the same way, but using SNP genotypes rather than QTL genotypes, and estimated effects rather than true effects. Thus, from the estimates of additive (*â*) and dominance effects ($\widehat{d}$), the GEBVP (for purebred performance) for animal *i* from breed *r* was calculated as:

2 $$ GEBV{P}_{ir}={\displaystyle \sum_{j=1}^{1000}}\left[\left({x}_{ij}\right)\left({p}_{jr}{\widehat{a}}_j+{q}_{jr}{\widehat{d}}_j\right)\right]+\left[\left({y}_{ij}\right)\left(0.5{p}_{jr}{\widehat{a}}_j+0.5{q}_{jr}{\widehat{d}}_j+0.5{p}_{jr}{\widehat{d}}_j-0.5{q}_{jr}{\widehat{a}}_j\right)\right]+\left[\left({z}_{ij}\right)\left(-{q}_{jr}{\widehat{a}}_j+{p}_{jr}{\widehat{d}}_j\right)\right]. $$

For the calculation of GEBVC (for crossbred performance), SNP frequencies in the other breed were used i.e. *p*_*jr*_ and *q*_*jr*_ in Equation 2 were replaced by *p*_*jŕ*_ and *q*_*jŕ*_ where *p*_*jŕ*_ and *q*_*jŕ*_ are the allele frequencies (A and a) for the *j*^*th*^ marker in breed *r’.* SNP frequencies in the other breed were calculated based on marker genotypes of all selection candidates in that breed.

### Accuracies of additive and dominance effects

In order to evaluate the accuracy of estimated additive and dominance effects separately, both true and estimated breeding values of an individual were partitioned into components of additive and dominance effects. For example, according to Equation 1, the TBV of an individual *i* is a function of additive effects, dominance effects and allele frequencies, and can be written as *TBV*_*i*_ = ∑*TBV*_*Add*_ + ∑*TBV*_*Dom*_, where ∑*TBV*_*Add*_ is the component of the TBV of animal *i* that is due to additive effects, and ∑*TBV*_*Dom*_ is the component of the TBV of animal *i* that is due to dominance effects. Equations 3 and 4 show the calculation of the TBV due to additive and dominance effects for animal *i* respectively:

3 $$ TB{V}_{Add}={\displaystyle \sum_{j=1}^{100}}\left[\left({x}_{ij}\right)\left({p}_{jr}{a}_j\right)\right]+\left[\left({y}_{ij}\right)\left(0.5{p}_{jr}{a}_j-0.5{q}_{jr}{a}_j\right)\right]+\left[\left({z}_{ij}\right)\left(-{q}_{jr}{a}_j\right)\right] $$

And

4 $$ TB{V}_{Dom}={\displaystyle \sum_{j=1}^{100}}\left[\left({x}_{ij}\right)\left({q}_{jr}{d}_j\right)\right]+\left[\left({y}_{ij}\right)\left(0.5{q}_{jr}{d}_j+0.5{p}_{jr}{d}_j\right)\right]+\left[\left({z}_{ij}\right)\left({p}_{jr}{d}_j\right)\right] $$

Symbols used in Equations 3 and 4 are the same as in Equation 1. Similarly, the GEBV of an individual *i* was calculated as *GEBV*_*i*_ = ∑*GEBV*_*Add*_ + ∑*GEBV*_*Dom*_, where ∑*GEBV*_*Add*_ and ∑*GEBV*_*Dom*_ are the components of the estimated breeding value of animal *i* due to estimated additive and dominance effects, respectively. GEBV due to additive and dominance effects were calculated in the same way as in Equations 3 and 4, but using SNP genotypes rather than QTL genotypes, and estimated effects rather than true effects. After partitioning the breeding value of each individual, the accuracy of estimated additive effects was calculated as the correlation between the TBV due to additive effects (*TBV*_*Add*_ ) and the GEBV due to additive effects (*GEBV*_*Add*_ ). Similarly, the accuracy of estimated dominance effects was calculated as the correlation between the TBV due to dominance effects (*TBV*_*Dom*_ ) and the GEBV due to dominance effects (*GEBV*_*Dom*_ ).

### Scenarios

Response to selection in CP was examined in five scenarios (Table 2). Simulated scenarios differed in structure of the training population and also in the criterion of selection. In all scenarios, breed A acted as the sire breed and breed B acted as the dam breed. In the reference scenario, both pure lines were selected for purebred performance, and both pure lines had their own reference population. In all other scenarios, breed A was selected for CP. Selection in breed B was for purebred performance in scenarios 1 and 3, and for CP in scenarios 2 and 4. In scenarios 1 and 2, both populations had their own reference population, while the reference population was combined in scenarios 3 and 4. In order to increase resolution between scenarios, we used the same population simulated from step 1 to step 3 (Figure 1) for a given replicate of each scenario. Each scenario was replicated 30 times.

**Table 2:** **Simulated scenarios**

**Scenarios**	**Selection criterion**		**Training population structure**
	**Breed A**	**Breed B**	
Reference scenario	GEBVP	GEBVP	Separate
Scenario 1	GEBVC	GEBVP	Separate
Scenario 2	GEBVC	GEBVC	Separate
Scenario 3	GEBVC	GEBVP	Common
Scenario 4	GEBVC	GEBVC	Common

GEBVP: selection in purebred breeds A and B is based on genomic estimated breeding value for purebred performance; GEBVC: selection in purebred breeds A and B is based on genomic estimated breeding value for crossbred performance; separate training means that each breed had its own training set; common stands for the combination of animals from breeds A and B to estimate marker effects.

We compared our scenarios under two conditions, i.e. low and high correlation of LD phase between the two breeds. In order to increase the correlation of LD phase between the two breeds, we increased LD in the common ancestral population by decreasing effective population size. Sved et al. [18] showed that, if two populations diverge from a common ancestral population, their correlation of LD phase is approximately equal to *r*_0_^2^(1 − *c*)^2*T*^, where *r*_0_^2^ is LD in the common ancestral population, *c* is the recombination rate between markers, and *T* is the time since breed divergence in generations.

### LD and correlation of LD phase

To evaluate the extent and magnitude of LD in the training populations and its impact on accuracy, LD was measured by *r*^2^ [19]. Only markers with a MAF greater than 0.1 were considered in this analysis, because the power of detection of LD between two loci is minimal when at least one of the loci has an extreme allele frequency [20]. To determine the decay of LD with increasing distance between SNPs, the average *r*^2^ within each breed was expressed as a function of distance between SNPs. SNP pairs were grouped by their pairwise distance into intervals of 1 cM, starting from 0 up to 100 cM. The average *r*^2^ for SNP pairs in each interval was estimated as the mean of all *r*^2^ within that interval.

To estimate persistence of LD phase between two breeds, only segregating SNPs (MAF > 0) in both breeds were included in the analysis. Persistence of LD phase was estimated following Badke et al. [21] as:

$$ {R}_{AB}=\frac{{\displaystyle {\sum}_{\left(i,j\right)\in p}}\left({r}_{ij(A)}-{\overline{r}}_A\right)\left({r}_{ij(B)}-{\overline{r}}_B\right)}{sd(A)sd(B)}, $$

where *R*_*A*,*B*_ is the correlation between *r*_*ij*(*A*)_ in breed A and *r*_*ij*(*B*)_ in breed B, *sd(A)* and *sd(B)* are the standard deviations of *r*_*ij*(*A*)_ and *r*_*ij*(*B*)_, respectively, and ${\overline{r}}_A$ and ${\overline{r}}_B$ are the average *r*_*ij*_ across all SNPs *i* and *j* within interval *p* for breeds A and B, respectively. Correlation of LD between the two lines was estimated for intervals of 1 cM (from 0 to 50 cM). SNPs with a pairwise distance greater than 50 cM were excluded since estimates of average *r*^2^ at greater distances are close to 0, which would result in the correlation of LD phase to be close to 0 as well.

## Results

### Distribution of marker allele frequencies

**Figure 2:**
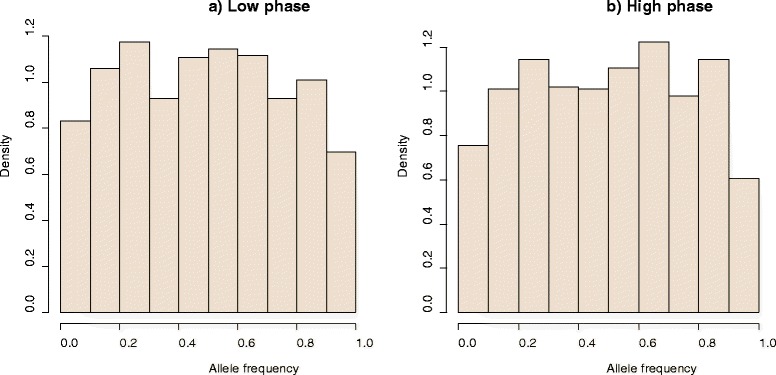
**Distribution of allele frequencies in the last generation of the historical population for a low (a) and a high correlation of LD phase (b).** The bounds are 0.01 and 0.99. The plots are the result of one replicate.

Figure 2 shows the distribution of marker allele frequencies for the last generation of the historical population. Since the initial allele frequencies were sampled from a uniform distribution, a kind of uniform distribution was expected with some fluctuations after 2000 generations of random mating, under a balance between mutation and random genetic drift due to finite population size. Although, a U-shaped distribution is typically observed with sequence data [22], allele frequencies on SNP chips tend to be uniform [23].

### Linkage disequilibrium

**Figure 3:**
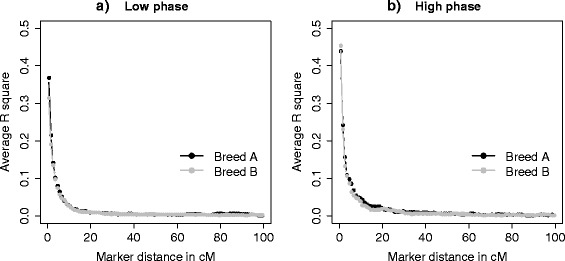
**Decay of average** ***r*** ^**2**^ **over distance for a low (a) and a high correlation of LD phase (b).** Average *r* ^2^ between SNPs in breed A and breed B at various distances in base pairs ranging from 1 to 100 cM. The plots are the result of one replicate.

To estimate LD, we used SNP genotypes of animals in the training set of both breeds. An average *r*^2^ of 0.43 and 0.42 for adjacent SNPs was found for breeds A and B, respectively. These average *r*^2^ between adjacent SNPs are similar to those reported by Badke et al. [21] for four US pig breeds that ranged from 0.36 to 0.46 for animals genotyped using the Illumina PorcineSNP60 (number of markers M = 62 163). Another study by Du et al. [24] that investigated the range and extent of LD in six commercial pig lines (two terminal sire lines and four maternal lines) for 4500 autosomal SNPs, reported an average *r*^2^ of 0.2 and 0.07 for all pairs of SNPs that were approximately 1 and 5 cM apart, respectively, whereas we found average *r*^2^ of 0.29 and 0.08 at those distances. Figure 3 displays an overview of the decline of *r*^2^ over distance in both breeds. As expected, in both breeds the most tightly linked SNP pairs had the highest average *r*^2^, and the observed average *r*^2^ decreased rapidly as the map distance increased.

### Persistence of LD phase

**Figure 4:**
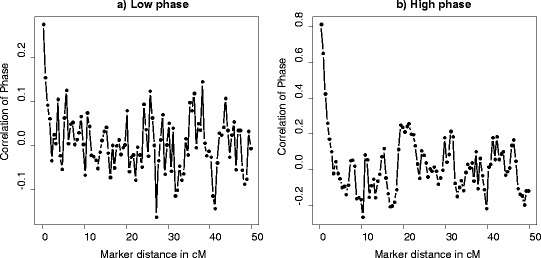
**Correlation of gametic phase compared across two breeds over distance for a low (a) and a high correlation of LD phase (b).** Correlation of LD phase between the two breeds for SNP pairs grouped by distance in intervals of 1 cM and covering 0 to 50 cM across the genome. The plots are the result of one replicate.

Persistence of LD phase among breeds can be used to infer on the history of a species and relatedness of breeds within that species, as well as on the reliability of across-population prediction of genome-wide association studies (GWAS) and GEVB [25]. Figure 4 shows the persistence of LD phase between adjacent SNPs, measured by the correlation of *r* between the two breeds. A greater correlation implies that the SNP-SNP (and most probably the SNP-QTL) LD is more consistent between the two breeds. As distance in time between subpopulations increases, there is a greater chance for recombination to break down the LD that was present in the ancestral population and for drift to create new LD within each subpopulation. Both mechanisms decrease the correlation of LD phase between the two breeds [26,27]. For SNPs with a pairwise distance of 1 cM, persistence of LD phase between breeds A and B was estimated 0.2 and 0.7 for cases with a low and high correlation of LD phase, respectively. Persistence of LD phase has been reported for Duroc, Landrace, Yorkshire pig breeds. For SNPs with a pairwise distance less than 50 kb, Badke et al. [21] reported a correlation of LD of 0.85 between Landrace and Yorkshire breeds and of 0.82 between Duroc and Landrace and between Duroc and Yorkshire breeds. Assuming 1 cM is approximately 1 Mb, we found correlations of LD phase equal to 0.38 and 0.87 for SNPs with a pairwise distance less than 50 kb for cases with low and high correlations of LD phase between two breeds, respectively. The correlation of LD phase between pig breeds in different studies ranged from 0.80 to 0.92 for SNPs with a pairwise distance less than 10 kb. In a study on the extent and persistence of LD phase in Holstein-Friesian, Jersey, and Angus cattle, de Roos et al. [25] reported a correlation of LD phase that ranged from 0.7 to 0.97 between two breeds for SNPs with a pairwise distance less than 10 kb and a decline of this correlation as the distance between SNPs or divergence between breeds increased. In our study, as distance between SNPs increased, the correlation of LD phase between the two breeds decreased (0.5 at an average pairwise SNP distance of 1 cM). It has been reported that, while correlation of LD phase is similar for pig breeds and dairy cattle at short distance ranges (<10 kb), pig breeds generally show greater correlations of LD phase than dairy cattle at larger SNP distances [21].

### Response to selection in crossbred animals

**Figure 5:**
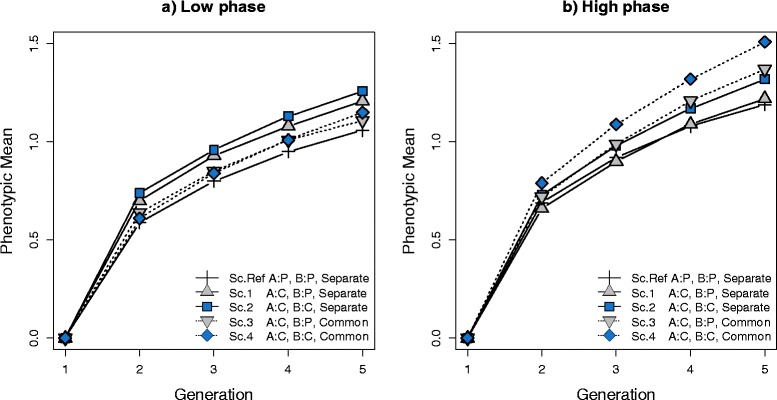
**Mean phenotype of crossbred individuals. (a)** Results for a low correlation of LD phase between breeds A and B (*r* = 0.2 for markers 1 cM apart). **(b)** Results for a high correlation of LD phase between breeds A and B (*r* = 0.7 for markers 1 cM apart). The plotted responses are means from 30 replicates. **Sc. Ref:** Selection criteria in both breed A and B was for purebred performance (P) and both breeds had **Separate** training sets. **Sc.1:** Selection criteria in breed A was for crossbred performance (C) and selection criteria in breed B was for purebred performance and both breeds had separate training sets. **Sc.2:** Selection criteria in both breed A and B was for crossbred performance and both breeds had separate training sets. **Sc.3:** Selection criteria in breed A was for crossbred performance and selection criteria in breed B was for purebred performance and both breeds had a **Common** training sets. **Sc.4:** Selection criteria in both breed A and B was for crossbred performance and both breeds had a common training set. Standard error of phenotypic means for simulated scenarios in generation 5 ranged from 0.03 to 0.04.

The purebred-crossbred genetic correlation, i.e. the correlation between TBVP and TBVC (*r*_*tbvp*,*tbvc*_), was equal to 0.66 and 0.70 on average for low and high correlations of LD phase, respectively. Figure 5 shows the mean values of phenotypes for crossbred animals in five generations under the five simulated scenarios with either a low (*r* = 0.2 in 1 cM) or a high correlation of LD phase (*r* = 0.7 in 1 cM) between the two breeds. When the correlation of LD phase was low between the two breeds, the ranking of scenarios in terms of mean phenotype of crossbred animals shows that breeding for CP led to higher gains in crossbred animals. By generation 5, scenario 2, in which both breeds were selected for CP, had a higher mean phenotype in the crossbred offspring than other scenarios. Scenario 1 also resulted in higher gain than the reference scenario since, in this scenario, one of the breeds was selected for CP. In the reference scenario, in which both breeds were selected for purebred performance, response to selection was lower than the other scenarios. Graph *a* in Figure 5 shows that, when each breed had a separate training set to estimate marker effects (scenarios 1 and 2), the performance of their crossbred offspring improved compared to that with the alternative scenarios for which a common reference was used to estimate marker effects (scenarios 3 and 4). For example, although in scenarios 1 and 3 one of the breeds (breed A) was selected for CP and because in scenario 1 each breed had its own training set, the response for scenario 1 was greater than for scenario 3.

In addition, when the correlation of LD phase was high between the two breeds, selection for CP improved the response in crossbred animals and the use of a combined reference population of the two breeds improved response even more. For scenarios 3 and 4, response in crossbred animals was greater than for the other scenarios, since these scenarios used a common training set to estimate marker effects.

### Heterosis in crossbred animals

**Figure 6:**
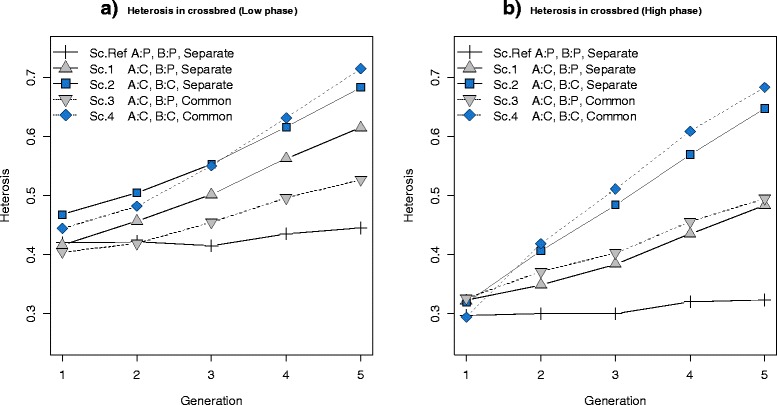
**Heterosis in crossbred individuals. (a)** Results for a low correlation of LD phase between breeds A and B (*r* = 0.2 for markers 1 cM apart). **(b)** Results for a high correlation of LD phase between breeds A and B (*r* = 0.7 for markers 1 cM apart). The plotted heterosis values are means from 30 replicates. **Sc. Ref:** Selection criteria in both breed A and B was for purebred performance (P) and both breeds had **Separate** training sets. **Sc.1:** Selection criteria in breed A was for crossbred performance (C) and selection criteria in breed B was for purebred performance and both breeds had separate training sets. **Sc.2:** Selection criteria in both breed A and B was for crossbred performance and both breeds had separate training sets. **Sc.3:** Selection criteria in breed A was for crossbred performance and selection criteria in breed B was for purebred performance and both breeds had a **Common** training set. **Sc.4:** Selection criteria in both breed A and B was for crossbred performance and both breeds had a common training set.

Based on the definition of heterosis, expected CP can be written as CP = BA + H, where BA denotes the breed average of pure lines and H the heterosis present in the crossbred animals. Thus, the observed advantage of selection for CP in some scenarios may be due to greater response in BA or in H, or in both. Heterosis was calculated at each generation of the crossbred population (Figure 6) and Table 3 shows BA values for each scenario. Since heterosis was simulated due to dominance, total heterosis was simply the sum of heterosis at each locus, *H* = ∑ *d*_*l*_(*p*_*A*,*l*_ − *p*_*B*,*l*_)^2^, where *d*_*l*_ is the dominance effect at QTL *l*, *p*_*A*,*l*_ is the allele frequency at QTL *l* in breed A, and *p*_*B*,*l*_ is the allele frequency at QTL *l* in breed B [7]. For both low and high correlations of LD phase, the amount of heterosis in the reference scenario was constant over generations but in other scenarios in which at least one breed was selected for CP, the amount of heterosis increased in each generation, which indicates that selection for CP resulted in greater heterosis and finally in improved performance of crossbred animals. Since heterosis depends on the difference in allele frequencies between the two breeds, these results suggest that selection for CP moves allele frequencies in the two breeds in opposite directions and causes divergence in allele frequencies between both breeds.

**Table 3:** **Mean phenotypic average of breeds A and B in simulated scenarios**

	**Low correlation of LD phase**					**High correlation of LD phase**				
**G**	**Sc. Ref**	**Sc. 1**	**Sc. 2**	**Sc. 3**	**Sc. 4**	**Sc. Ref**	**Sc. 1**	**Sc. 2**	**Sc. 3**	**Sc. 4**
1	1.33	1.25	1.33	1.21	1.37	1.12	1.19	1.04	1.04	0.93
2	1.97	1.88	1.94	1.79	1.96	1.81	1.84	1.68	1.71	1.60
3	2.02	2.04	2.11	1.96	2.12	2.04	2.03	1.86	1.90	1.80
4	2.32	2.14	2.21	2.07	2.20	2.18	2.17	1.97	2.12	1.95
5	2.40	2.21	2.28	2.15	2.26	2.29	2.24	2.03	2.23	2.05

G = generation; Sc. Ref = reference scenario; Sc. 1 = scenario 1; Sc. 2 = scenario 2; Sc. 3 = scenario 3; Sc. 4 = scenario 4.

### Accuracy of selection

**Figure 7:**
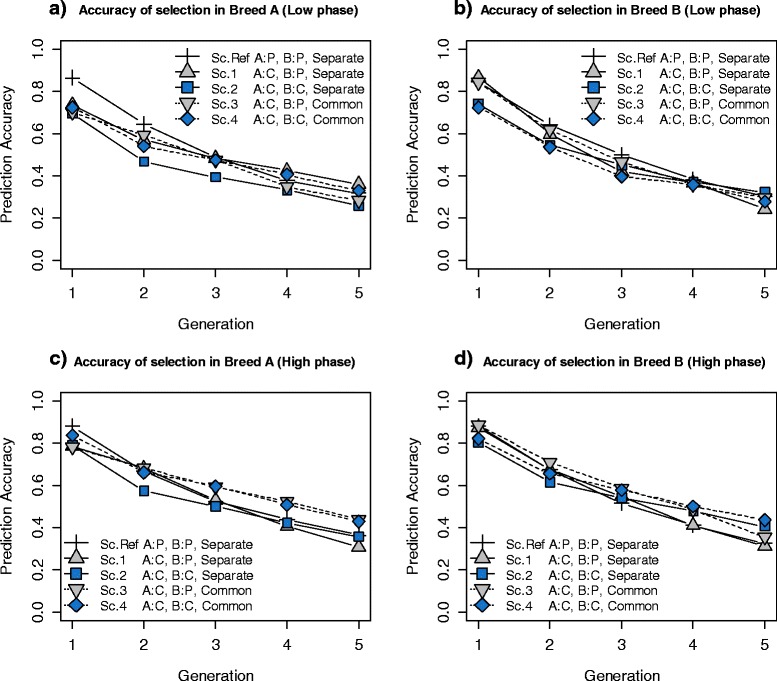
**Accuracy of selection in breeds A and B in five scenarios. (a)** and **(b)** Results for a low correlation of LD phase between breeds A and B (*r* = 0.2 for markers 1 cM apart) **(c)** and **(d)** Results for a high correlation of LD phase between breeds A and B (*r* = 0.7 for markers 1 cM apart). The plotted accuracies are means from 30 replicates. **Sc. Ref:** Selection criteria in both breed A and B was for purebred performance (P) and both breeds had **Separate** training sets. **Sc.1:** Selection criteria in breed A was for crossbred performance (C) and selection criteria in breed B was for purebred performance and both breeds had separate training sets. **Sc.2:** Selection criteria in both breed A and B was for crossbred performance and both breeds had separate training sets. **Sc.3:** Selection criteria in breed A was for crossbred performance and selection criteria in breed B was for purebred performance and both breeds had a **Common** training set. **Sc.4:** Selection criteria in both breed A and B was for crossbred performance and both breeds had a common training set. It should be noted that accuracies in this Figure are correlations between the selection criterion and the EBV of interest. Thus, when selection is for purebred performance, accuracy is the correlation between GEBVP and TBVP, while when selection is for crossbred performance, accuracy is the correlation between GEBVC and TBVC.

Prediction accuracy, i.e. correlation between the breeding values predicted by GS and the TBV obtained from simulation, ranged from 0.69 to 0.86 in the validation population (generation 1) across the different scenarios analysed (Figure 7). It should be noted that accuracies in Figure 7 always refer to the selection criterion. In other words, when selection is for purebred performance, accuracy is the correlation between TBVP and GEBVP, i.e. (*r*_*tbvp*,*gebvp*_). Conversely, when selection is for CP, accuracy is the correlation between TBVC and GEBVC, i.e. (*r*_*tbvc*,*gebvc*_). Hence, this comparison shows whether selection for CP is, or is not, more difficult than selection for purebred performance.

For a low correlation of LD phase, Figures 7a and 7b show that accuracy of selection for breed A was greater in the reference scenario (in which breed A was selected for purebred performance) than in the other scenarios (in which breed A was selected for CP. Accuracy of selection in breed B (Figure 7b) was also greater when selection in this breed was for purebred performance (reference scenario and scenarios 1 and 3) than when selection was for CP (scenarios 2 and 4). Thus, predicting GEBVC based on purebred data is more difficult than predicting GEBVP on such data.

For a high correlation of LD phase (Figure 7c and 7d), accuracies ranged from 0.78 to 0.88 in the first generation, which suggests that when the correlation of LD phase between breeds is high, there is a smaller difference in accuracy between purebred and crossbred selection (*r*_*tbvp*,*gebvp*_ ~ *r*_*tbvc*,*gebvc*_). Finally, for both low and high correlations of LD phase, prediction accuracy declined over generations in all scenarios.

### Accuracies of additive and dominance effects

The accuracies reported above are correlations between TBV and GEBV and include both additive and dominance components of the breeding values *per se*. In order to compare the accuracy of estimates of additive and dominance effects separately, both true and estimated breeding values of an individual were partitioned into components due to additive and dominance effects. Table 4 includes accuracies of estimated breeding values, as well as accuracies of the additive and dominance components of estimated breeding values for low and high correlations of LD phase between the two breeds. It should be noted that accuracies of estimated breeding values in Table 4 always refer to the selection criterion. In other words, when selection in a breed is for purebred performance, accuracy is the correlation between TBVP and GEBVP. Conversely, when selection in a breed is for CP, accuracy is the correlation between TBVC and GEBVC. Generally, in all scenarios, accuracies of estimated breeding values due to additive effects were greater than accuracies of estimated breeding values due to dominance effects. These differences in accuracies were clearer for scenarios in which selection within a breed was for CP (e.g. breed B in scenarios 2 and 4 in Table 4). However, when selection in a breed was for purebred performance, accuracies of estimated breeding values due to additive and dominance effects were not very different (e.g. breed B in the reference scenario and scenarios 1 and 3). In summary, for both selection criteria, accuracies of estimated breeding values were as high as accuracies due to additive effects. However, when selection within a breed was for purebred performance, accuracies due to dominance effects were higher than accuracies due to dominance effects for selection on CP. The same trend was observed with a high correlation of LD phase between the two breeds [See Additional file 1].

**Table 4:** **Partitioning accuracies of breeding values due to additive and dominance effects for a low correlation of LD phase**

		**Ref scenario**			**Scenario 1**			**Scenario 2**			**Scenario 3**			**Scenario 4**		
Breed A	G	**BV**	**Add**	**Dom**	**BV**	**Add**	**Dom**	**BV**	**Add**	**Dom**	**BV**	**Add**	**Dom**	**BV**	**Add**	**Dom**
	1	0.86	0.81	0.53	0.73	0.80	0.22	0.69	0.80	0.15	0.70	0.78	0.26	0.72	0.76	0.31
	2	0.64	0.69	0.56	0.57	0.65	0.20	0.46	0.69	0.19	0.59	0.69	0.27	0.54	0.65	0.22
	3	0.48	0.63	0.57	0.48	0.50	0.23	0.39	0.63	0.20	0.47	0.61	0.21	0.47	0.61	0.22
	4	0.37	0.59	0.60	0.42	0.52	0.24	0.33	0.57	0.21	0.34	0.54	0.18	0.40	0.58	0.24
	5	0.31	0.56	0.61	0.36	0.47	0.23	0.25	0.52	0.20	0.28	0.48	0.20	0.32	0.52	0.26
		**Ref scenario**			**Scenario 1**			**Scenario 2**			**Scenario 3**			**Scenario 4**		
Breed B	G	**BV**	**Add**	**Dom**	**BV**	**Add**	**Dom**	**BV**	**Add**	**Dom**	**BV**	**Add**	**Dom**	**BV**	**Add**	**Dom**
	1	0.85	0.77	0.47	0.87	0.81	0.56	0.74	0.81	0.13	0.88	0.85	0.60	0.72	0.82	0.19
	2	0.64	0.65	0.43	0.60	0.64	0.55	0.55	0.68	0.16	0.71	0.76	0.59	0.54	0.69	0.18
	3	0.50	0.58	0.49	0.42	0.59	0.55	0.45	0.59	0.18	0.59	0.70	0.63	0.40	0.62	0.16
	4	0.38	0.58	0.53	0.37	0.56	0.54	0.37	0.54	0.19	0.49	0.65	0.68	0.36	0.56	0.15
	5	0.30	0.55	0.56	0.24	0.54	0.58	0.32	0.49	0.18	0.35	0.60	0.68	0.28	0.48	0.14

Reference scenario = selection criteria in both breeds A and B were for purebred performance (P) and both breeds had each a separate training set; scenario 1 = selection criteria in breed A was for crossbred performance (C) and selection criteria in breed B was for purebred performance and both breeds had each a separate training set; scenario 2 = selection criteria in both breeds A and B were for crossbred performance and both breeds had each a separate training set; scenario 3 = selection criteria in breed A was for crossbred performance and selection criteria in breed B was for purebred performance and both breeds had a common training set; scenario 4 = selection criteria in both breeds A and B were for crossbred performance and both breeds had a common training set.

### Response to selection in purebred animals

**Figure 8:**
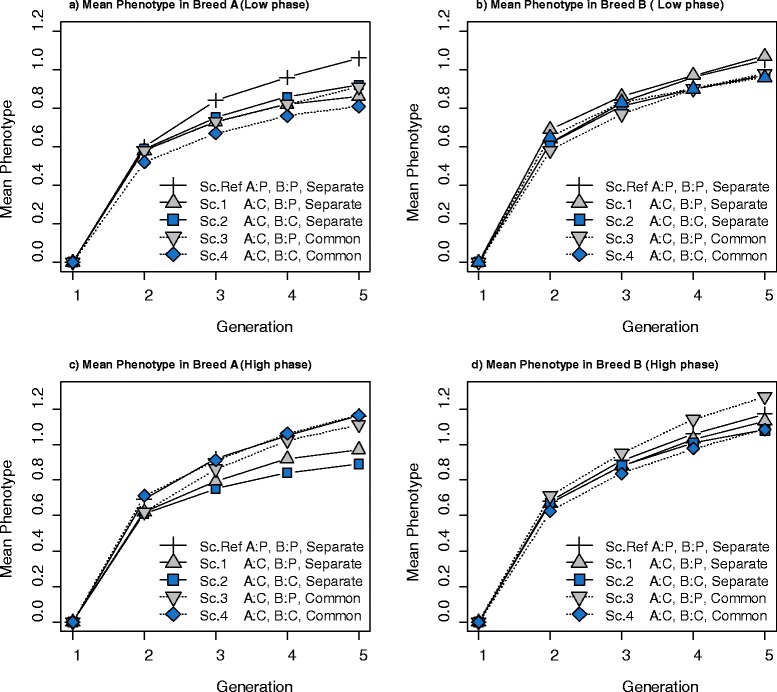
**Mean phenotype of purebred individuals. (a)** and **(b)** Results for a low correlation of LD phase between breeds A and B (r = 0.2 for markers 1 cM apart)**. (c)** and **(d)** Results for a high correlation of LD phase between breeds A and B (r = 0.7 for markers 1 cM apart). The plotted responses are means from 30 replicates. **Sc. Ref:** Selection criteria in both breed A and B was for purebred performance (P) and both breeds had **Separate** training sets. **Sc.1:** Selection criteria in breed A was for crossbred performance (C) and selection criteria in breed B was for purebred performance and both breeds had separate training sets. **Sc.2:** Selection criteria in both breed A and B was for crossbred performance and both breeds had separate training sets. **Sc.3:** Selection criteria in breed A was for crossbred performance and selection criteria in breed B was for purebred performance and both breeds had a **Common** training set. **Sc.4:** Selection criteria in both breed A and B was for crossbred performance and both breeds had a common training set.

Figure 8 shows the response to selection in both purebred populations of breeds A and B over five generations. For a low correlation of LD phase between breeds A and B (Figures 8a and 8b), response to selection in both breeds in the reference scenario was higher than in the other scenarios, since selection in this scenario was for purebred performance. In the other scenarios, response to selection was lower for breed A than in the reference scenario, since in these scenarios the selection criterion was CP (Figure 8a). Figure 8b shows that response to selection for breed B in scenarios 3 and 4, which used a common reference population, was lower than in the other scenarios.

For a high correlation of LD phase between breeds A and B, response to selection for breed A was lower in scenario 2 than in the other scenarios (Figure 8c). Figure 8c also shows that for a high correlation of LD phase between breeds, the use of a common reference population to estimate marker effects improved the performance of purebred animals, i.e. scenario 3 performed better than scenario 1, and scenario 4 performed better than scenario 2.

In conclusion, for both low and high correlations of LD phase, selection for CP generated a loss in response to selection in purebred animals.

## Discussion

The purpose of this study was to evaluate the potential benefit of GS within purebred lines, when the objective is to improve performance of crossbred populations at the commercial level and phenotypic information is collected only on purebred animals. We compared response to selection in crossbred animals in five scenarios, where individuals were selected either on GEBVP or GEBVC, and marker effects were estimated either from two separate purebred reference populations or a combined purebred reference population. In a two-way crossbreeding system, we found that selection for GEBVC increased response in crossbred animals compared to selection for GEBVP. We also investigated the effect of the correlation of LD phase between the two pure breeds on the consequences of combining both reference populations. The results revealed that, for a high correlation of LD phase, combining both populations into a single reference population increased response to selection in crossbred animals.

### Persistence of LD phase

The value of SNPs effect estimated for populations other than the reference population depends on the persistence of LD phase between the reference population and the other population [28]. For example, a SNP that was identified as being in LD with the QTL in one breed may not be in LD with the QTL in another breed. The level of LD is more likely to be different between two populations when these populations have diverged for many generations and the effective population size becomes small, and when distance between the SNP and the QTL is large, since these factors will either break down LD in the ancestral population or create new LD within the subpopulation [3,26].

For a low correlation of LD phase, combining data from both breeds to estimate marker effects (scenarios 3 and 4) had no effect on the accuracy of GS. It has been reported that using multiple breeds to predict GEBV can be effective to increase the size of the reference population and in turn increase accuracy of selection [29]. However, the benefit of combining reference populations depends on the size of the reference population, since there is a diminishing return relationship between size and accuracy of reference populations. Hence, if the reference population is small, combining populations may help when the correlation of LD phase is sufficiently high but will have a limited benefit or may even be detrimental when the reference population is large.

For a high correlation of LD phase, combining animals from the two breeds in the training set improved the accuracy of selection in scenarios 3 and 4. These results are consistent with those of de Roos et al. [30], who concluded that across-population evaluations were preferred to within-population evaluations when the populations were closely related, marker density was high, or the number of animals with phenotypic records was small.

### Non-additive effects and response to selection

It has been argued that dominance is the likely genetic basis of heterosis [7], therefore explicitly including dominance in the GS model may be an advantage when selecting purebred animals for CP, i.e. it may increase heterosis. In this study, we assumed dominance variance to be one third of the additive genetic variance. This ratio resulted in 10 to 15% of loci showing overdominance. When overdominance is present, crossbred performance is maximized if alternate alleles are fixed in the two purebred populations. In fact with overdominance, allele substitution effects may have opposite signs in the parental breeds, depending on allele frequencies in the two breeds. In this case, the two parental breeds are expected to be fixed for alternate alleles of overdominant QTL, which increases the frequency of favourable heterozygotes in crossbred progeny and can explain the benefit of selection based on GEBVC. However, it should be noted that existence of overdominance is not the only driver of divergence in allele frequencies in parental breeds. It has been shown that partial dominance can play a role in influencing changes in allele frequencies and have favourable effects on heterosis, especially when the number of QTL that affect the trait is large [31].

### Genotype-by-environment and genotype-by-genetic interactions

In our simulation, we assumed that the additive and dominance effects of the QTL alleles were similar in both breeds. For some QTL, which have been traced to known mutations, the alleles do act reasonably similarly in different breeds and populations [32]. However, this assumption is violated when there are QTL-by-environment interactions or QTL-by-genetic background interactions (epistasis). With substantial QTL-by-environment interactions or epistasis, it will be less advantageous to combine populations in a training set, because marker effects will differ between populations [30]. In addition, with genotype-by-environment (G × E) interaction and epistasis, the main complication is that the dominance model does not fully explain the incomplete genetic correlation between crossbred and purebred individuals (*r*_*pc*_). In fact, an incomplete genetic correlation between purebred and crossbred performance can be due to both non-additive effects (dominance and epistasis), and G × E interaction. In our simulation, the correlation between TBVP and TBVC (*r*_*tbvp*,*tbvc*_) was 0.66 and 0.7 on average for low and high correlations of LD phase between two breeds, respectively, which was purely due to dominance and differences in allele frequencies between the two purebred lines.

In this study, we focused on using purebred data to improve CP. In fact, selection at the purebred level reduces the need for the crossbred testing that is required for CCPS, thereby saving important test resources and enabling the short generation intervals of purebred selection. However, Dekkers and Chakraborty [33] discussed the benefit of GS for improving CP and suggested that it may be limited if marker effects are estimated from purebred nucleus data since the resulting EBV are strictly relevant to the studied population and environment only and may not help much to improve selection for CP if substantial G × E and genotype-by-genetic (G × G) background interactions are present. In this study, we considered the G × G due to dominance and not that due to differences in the physical environment. In principle, one could use a dominance model and multitrait analysis to partition the purebred-crossbred genetic correlation into a component due to dominance and a remaining component due to G × E and epistasis. However, accurate partitioning would require a small standard error of the estimated purebred-crossbred genetic correlation, and thus very large datasets [34].

In this study, directional dominance was simulated since dominance coefficients (*h*_*i*_) were normally distributed with a positive mean, *N*(0.5, 0.1). Consequently, dominance effects (*d*_*i*_) were on average greater than 0 (*d* > 0). However, in the statistical model used to estimate the genetic effects associated with each marker, dominance effects were considered as random unknown effects with a mean of 0. The simulation of dominance effects that are on average greater than 0 has two consequences. First, the overall average trait value may increase. This will be accounted for by the fixed effects component of the model *Xb*. Second, directional dominance leads to inbreeding depression. Thus, animals with different inbreeding levels will have systematically different trait phenotypes. This probably means that our model could be improved by including a regression on inbreeding coefficients. However, we think this effect is probably limited since we simulated only five discrete generations of data with random mating among selected animals. Thus, the range of inbreeding coefficients may not have been sufficiently large to affect the results.

## Conclusions

Under the hypothesis that crossbred animals differ from purebred animals because of dominance, GS can be applied to select purebred individuals for CP without collecting crossbred phenotypic or genotypic data, by using a dominance model. We found that in a two-way crossbreeding system, response to selection in crossbred individuals was higher when selection was for GEBV for CP, although data were collected on purebred individuals. Furthermore, if the correlation of LD phase between two breeds is high, there can be an added benefit in terms of accuracy of GEBV if animals from both breeds are combined into a single reference population to estimate marker effects.

## Additional files

Additional file 1:
**Partitioning accuracies of breeding values due to additive and dominance effects for a high correlation of LD phase.** Partitioning accuracies of breeding values due to additive and dominance effects for a high correlation of LD phase.
