# Supplementary material for: Maximizing crossbred performance through purebred genomic selection
Source: Genet Sel Evol. 2015 Mar 14;47(1):16. doi: 10.1186/s12711-015-0099-3 (PMC4358869; doi:10.1186/s12711-015-0099-3)
Supplement: Additional file 1: — Partitioning accuracies of breeding values due to additive and dominance effects for a high correlation of LD phase. Partitioning accuracies of breeding values due to additive and dominance effects for a high correlation of LD phase. [file 12711_2015_99_MOESM1_ESM.pdf]

Additional file 1:

Partitioning accuracies of breeding values due to additive and dominance effects for a high correlation of LD phase.

Partitioning accuracies of breeding values due to additive and dominance effects for a high correlation of LD phase

		Scenario Ref			Scenario 1			Scenario 2			Scenario 3			Scenario 4		
**Breed A**	**G**	**BV**	**Add**	**Dom**	**BV**	**Add**	**Dom**	**BV**	**Add**	**Dom**	**BV**	**Add**	**Dom**	**BV**	**Add**	**Dom**
	**1**	0.88	0.82	0.50	0.78	0.83	0.45	0.79	0.83	0.32	0.78	0.84	0.17	0.84	0.85	0.45
	**2**	0.67	0.71	0.53	0.68	0.75	0.46	0.57	0.69	0.32	0.68	0.75	0.35	0.66	0.72	0.49
	**3**	0.52	0.66	0.61	0.53	0.67	0.45	0.50	0.63	0.33	0.59	0.70	0.36	0.60	0.70	0.51
	**4**	0.44	0.63	0.65	0.41	0.62	0.44	0.42	0.58	0.34	0.52	0.66	0.39	0.51	0.65	0.52
	**5**	0.36	0.62	0.66	0.31	0.57	0.48	0.36	0.55	0.33	0.44	0.64	0.36	0.43	0.60	0.51
		**Scenario Ref**			**Scenario 1**			**Scenario 2**			**Scenario 3**			**Scenario 4**		
**Breed B**	**G**	**BV**	**Add**	**Dom**	**BV**	**Add**	**Dom**	**BV**	**Add**	**Dom**	**BV**	**Add**	**Dom**	**BV**	**Add**	**Dom**
	**1**	0.88	0.84	0.51	0.87	0.82	0.47	0.80	0.83	0.37	0.88	0.85	0.60	0.82	0.83	0.49
	**2**	0.67	0.71	0.55	0.68	0.70	0.51	0.62	0.73	0.42	0.71	0.76	0.59	0.65	0.75	0.41
	**3**	0.51	0.68	0.59	0.55	0.66	0.58	0.54	0.70	0.44	0.59	0.70	0.63	0.58	0.70	0.41
	**4**	0.41	0.64	0.63	0.41	0.60	0.64	0.48	0.64	0.41	0.49	0.65	0.68	0.50	0.65	0.39
	**5**	0.33	0.63	0.65	0.31	0.54	0.63	0.41	0.59	0.38	0.35	0.60	0.68	0.44	0.62	0.38

BV: Accuracy of breeding values that is correlation between the selection criterion and the EBV of interest. Thus, when selection is for purebred performance, accuracy is the correlation between GEBVP and TBVP, while when selection is for crossbred performance, accuracy is the correlation between GEBVC and TBVC. **Add:** Accuracy of breeding values due to additive effects. **Dom:** Accuracy of breeding values due to dominance effects. **G:** generation.

Scenario Ref: Selection criteria in both breed A and B was for purebred performance (P) and both breeds had Separate training set. **Scenario 1:** Selection criteria in breed A was for crossbred performance (C) and selection criteria in breed B was for purebred performance and both breeds had separate training set. **Scenario 2:** Selection criteria in both breed A and B was for crossbred performance and both breeds had separate training set. **Scenario 3:** Selection criteria in breed A was for crossbred performance and selection criteria in breed B was for purebred performance and both breeds had Common training set. **Scenario 4:** Selection criteria in both breed A and B was for crossbred performance and both breeds had common training set.
